# Resource potential and essential oil composition of *Artemisia arenaria* DC. in the Northern Aral Sea Region

**DOI:** 10.7717/peerj.21295

**Published:** 2026-05-13

**Authors:** Zhuldyz Salmukhanbetova, Zhanat Karzhaubekova, Liliya Dimeyeva, Nadezhda Gemejiyeva

**Affiliations:** 1 Institute of Botany and Phytointroduction, Almaty, Kazakhstan; 2 Al-Farabi Kazakh National University, Almaty, Kazakhstan

**Keywords:** *Artemisia arenaria*, Northern Aral Sea Region, Resource potential, Essential oil constituents, Yield, Operational stock

## Abstract

**Background:**

Communities dominated by * Artemisia arenaria* DC. are widely distributed in the sandy massifs of northern deserts of the Turan lowland, where they perform landscape-forming, relief-stabilising functions. Despite its ecological importance, *A. arenaria* remains insufficiently studied in Kazakhstan, in particular with regard to its phytochemical properties and raw material reserves in the Northern Aral Sea region. Therefore, this study focuses on the preliminary assessment of the resource potential and essential oil composition of the aboveground parts of *A. arenaria* in this region.

**Methods:**

Classical geobotanical methods were used to describe each site and its resource objects. The yield was determined using two basic methods: clipping and model bushes. Based on the obtained yield data, the operational stock and the volume of possible annual processed air-dried raw material were calculated, taking into account a four-year recovery period. Essential oil was isolated from the air-dried aerial parts of *A. arenaria* by hydrodistillation. The chemical composition of the essential oil was analysed using gas chromatography coupled with mass spectrometry (GC-MS).

**Results and Conclusion:**

The assessment of *A. arenaria* resource potential in the Northern Aral Sea region identified five thickets, covering an area of 51.8 ha. In these thickets, total operational stock was 13.06 tons of air-dried raw materials with a possible annual processed volume of 2.61 tons. GC-MS analysis of *A. arenaria* essential oil from aerial parts identified 105 compounds with mass spectral matches ≥ 85%. The oil was dominated by 18 compounds (approximately 70% of the total), with pseudolimonene (12.6%), α-bisabolol (11.77%), geranyl acetate (7.74%), and 3-carene (6.5%) as the major constituents. Several components detected in this study, including geranyl acetate, have not previously been reported for this species in the available literature. These findings expand current knowledge of the chemical composition of *A. arenaria* and may contribute to future investigations of its chemotypic variability and potential biological properties.

## Introduction

For more than 30 years, the Northern Aral Sea region has been classified as an ecological disaster zone (Law of the Republic of Kazakhstan, 1992). It occupies an extensive area in western Kazakhstan within the subzone of northern deserts of the temperate belt (Rachkovskaya et al., 2003), between 58°00′–62°00′ east longitude and 46°00′–48°00′ north latitude. The region’s climate is very dry and moderately hot, with pronounced atmospheric drought (Selyaninov Hydrothermal Index of 0.4–0.6) and annual precipitation of around 150 mm. The average air temperature is 8.3 °C, with absolute minima reaching −45 °C in winter and maxima up to +45 °C in summer. The relative air humidity varies markedly throughout the year, from approximately 40% in summer to 79–81% in winter (Bedarev, Gerasimenko & Korobova, 1971; Dolgikh, 2010; Gemejiyeva et al., 2025). Such extreme arid conditions strongly influence vegetation, shaping plant communities adapted to saline and arid habitats.

The area remains relatively underexplored regarding its plant resources, and there is no up-to-date information on the use and raw material base of economically valuable plant species. This gap complicates the identification of medicinal plants with diverse biological activity and the evaluation of their resource potential in a region severely affected by environmental degradation.

Within this ecological and climatic context, particular attention is given to the genus *Artemisia* L., a large and polymorphic genus comprising more than 500 plant species. Many species of the genus are widely used in folk medicine and complementary medicine systems (Mohammed, 2022; Mohammed et al., 2022), and are also used in modern medicine for the treatment of inflammatory diseases, gastrointestinal disorders, fever, malaria, hepatitis, and cancer (Mirbehbahani et al., 2020; Sadiq, Hayat & Ashraf, 2014; Jang et al., 2015). Additionally, they exhibit acaricidal, insecticidal, and repellent properties (Ushbaeva et al., 2000). Studies are being conducted worldwide to explore the therapeutic potential of representatives of this genus (Ashraf, Hayat & Mumtaz, 2010). The pharmacological activity of plants belonging to this genus is attributed to the presence of bitter compounds (azulene-type lactones), particularly essential oils (Abad et al., 2012; Nagy & Tengerdy, 1968), as well as sesquiterpenes, flavonoids, organic acids, coumarins, saponins and alkaloids (Bisht et al., 2021; Zeb et al., 2019; Egeubaeva, 2002; Atazhanova, 2008).

In addition to their medicinal significance, several species of the genus *Artemisia* have significant economic value, serving as food, fodder and ornamental plants, as well as functioning as soil stabilisers in disturbed ecosystems (Ashraf, Hayat & Mumtaz, 2010). They are also used as flavouring agents in perfumery and cosmetics, as natural dyes for wool, as food additives, and serve as melliferous plants (Budantsev & Lesiovskaya, 2001).

There are 86 species of the genus *Artemisia* in Kazakhstan (Baitenov, 2001), of which more than half have been studied for medicinal purposes. However, data on raw material resources are available only for 10 species (Grudzinskaya et al., 2014; Gemejiyeva et al., 2023). The remaining species, including *Artemisia arenaria* DC., present a promising opportunity for resource studies.

### Ecological distribution of *A. arenaria* communities

Communities dominated by *A. arenaria* are widely distributed across the sandy massifs of the northern deserts of the Turan lowland, forming stable, long-term communities across all edaphic sand variants (Rachkovskaya et al., 2003). Within this broad geographical range, psammophytic-wormwood communities dominated by *A. arenaria* are especially common in the west part of Kazakhstan, along the Caspian Sea coast and in the Aral regions, including the Bolshye and Malye Barsuki sandy massifs and the Aral Karakum sands. In the Northern Aral Sea region, within the sands of the Bolshye and Malye Barsuki, *A. arenaria* forms combinations with such communities as *Calligonum aphyllum-Agropyron fragile-Koeleria glauca*, which are distributed in both the hilly and honeycomb sands and also on barkhans and degraded sandy massifs (Rachkovskaya et al., 2003).

### Pastoral significance and forage value of *A. arenaria*

Given its extensive distribution across sandy habitats, *A. arenaria* is also common in pasture landscapes, where it is considered a forage plant, although it is only weakly grazed due to the presence of essential oils that reduce its palatability. Nevertheless, its chemical composition indicates a relatively high nutritional value. Annual shoots with leaves have the following contents (%): protein—10.8–14.9, fibre—16.4–30.8, fat—2.3−7.1, ash—6.5–12.0, nitrogen-free extractive substances—44.0–56.3 (Kurochkina, Osmanova & Karibaeva, 1986).

Despite its ecological importance, *A. arenaria* remains insufficiently studied in Kazakhstan with regard to its phytochemical properties, and its raw material reserves in the Northern Aral Sea region have not yet been assessed. Therefore, we selected *A. arenaria* DC. for this study.

We hypothesise that populations of *A. arenaria* in the Northern Aral Sea region are characterised by (1) considerable phytomass production and (2) a distinct essential oil profile, reflecting their phytochemical potential.

The objective of our research is to assess the resource potential and component composition of the essential oil from the aboveground part of *A. arenaria* raw material in the Northern Aral Sea region.

### Overview of phytochemical research on *A. arenaria*

Investigating *A. arenaria* is therefore justified, as prior studies have demonstrated that species in the subgenus *Dracunculus* (Bess.) Rydberg possesses diverse chemical constituents with relevance for medicinal applications. According to the Klyshev, Alyukina & Ryakhovskaya (1983), species of wormwood in the subgenus *Dracunculus* (cycle *Psammophilae*), to which *A. arenaria* belongs, are characterised by a high diversity of components and a high flavonoid content, ranging from 1.5 to 5.7%. A brief overview of the current research on the phytochemical properties of *A. arenaria* is provided below.

Velikorodov et al. (2011) investigated the chemical composition of the essential oil from *A. arenaria* in the Astrakhan region. Using GC-MS analysis, they identified 30 components, among which the dominant ones were α-bisabolol (34.5–34.9%), β-pinene (23.1–24.3%), α-pinene (7.9–8.4%), limonene (6.9−7.3%), camphor (5.2−5.4%), and 1,8-cineole (0.9−1.4%).

Furthermore, Ochkur et al. (2014) investigated the lipophilic extracts of *A. arenaria* growing in Crimea, identifying 10 carboxylic acids. Among them, the predominant ones were dicarboxylic unsaturated acids—succinic and malonic.

Chinese researchers (Guo et al., 1999) investigated the whole plants of *A. arenaria* and isolated two eudesmane-type sesquiterpenes (eudesmane and eudesm-5-ene-1β, 4α-diol), two common sterols (stigmasterol and β-sitosterol), two triterpenes (α- and β-amyrin), and five flavanoids (5, 7-dihydroxy-4′-methoxyflavanone, 5-hydroxy-7, 4′-dimethoxyflavanone 7,5,6,3′-trimethoxyflavanone, 5-hydroxy-7, 4′-dimethoxyflavanone, 5-hydroxy-6,7,4′-trimethoxyflavanone).

*Artemisia albicerata* Krasch. is now considered a synonym of *A. arenaria* according to the site Plants of the World Online (POWO) (Royal Botanic Gardens Kew, 2024). A quantitative analysis for its extract yield (12.71%), and for other biologically active substances showed the following data: alkaloids—8.30%; saponins—7.76%; flavonoids—7.26%; polysaccharides—1.44%; tannins—1.33%; coumarins—0.33%; and organic acids—0.32% (Amantay et al., 2019).

## Materials and Methods

### Plant material

**Figure 1:**
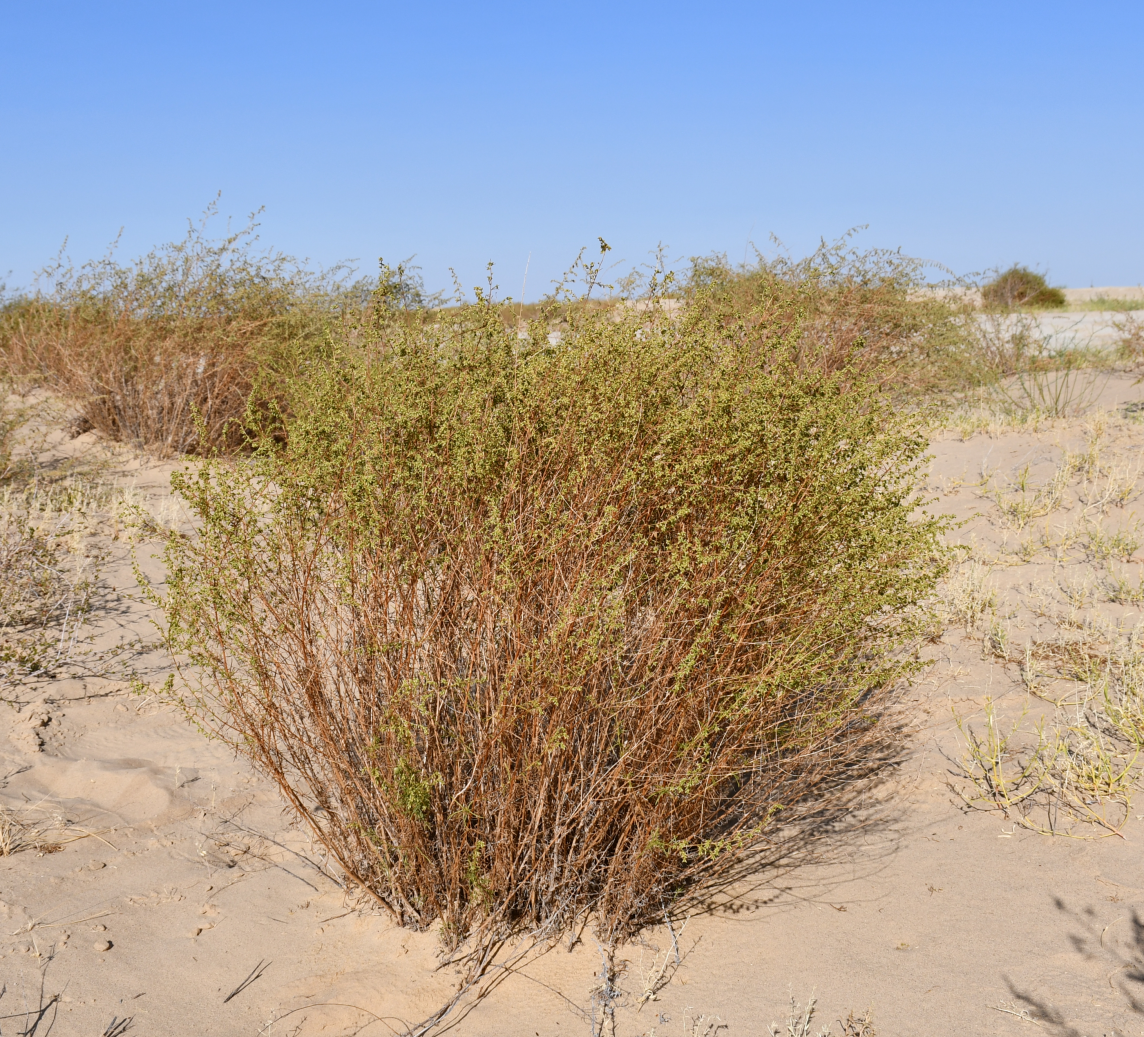
*Artemisia arenaria* on the sands of Bolshye Barsuki.

Sand wormwood *(A. arenaria* DC.) is a semi-shrub belonging to the *Asteraceae* family, with a height of 50–75 cm (occasionally reaching up to 180 cm), with a thick, woody, multi-headed root. Fruiting stems are numerous, erect, and ribbed. The lower stem leaves are petiolate, ovate in outline, 2–6 cm long and up to three cm wide, and twice pinnately dissected; the terminal leaf lobules are linear or narrowly linear, thickish, and acute at the apex. The middle stem leaves are sessile, with pinnately dissected lobes at the base. Flowering takes place in July–August (Fig. 1) (Pavlov, 1966).

### Data collection

**Figure 2:**
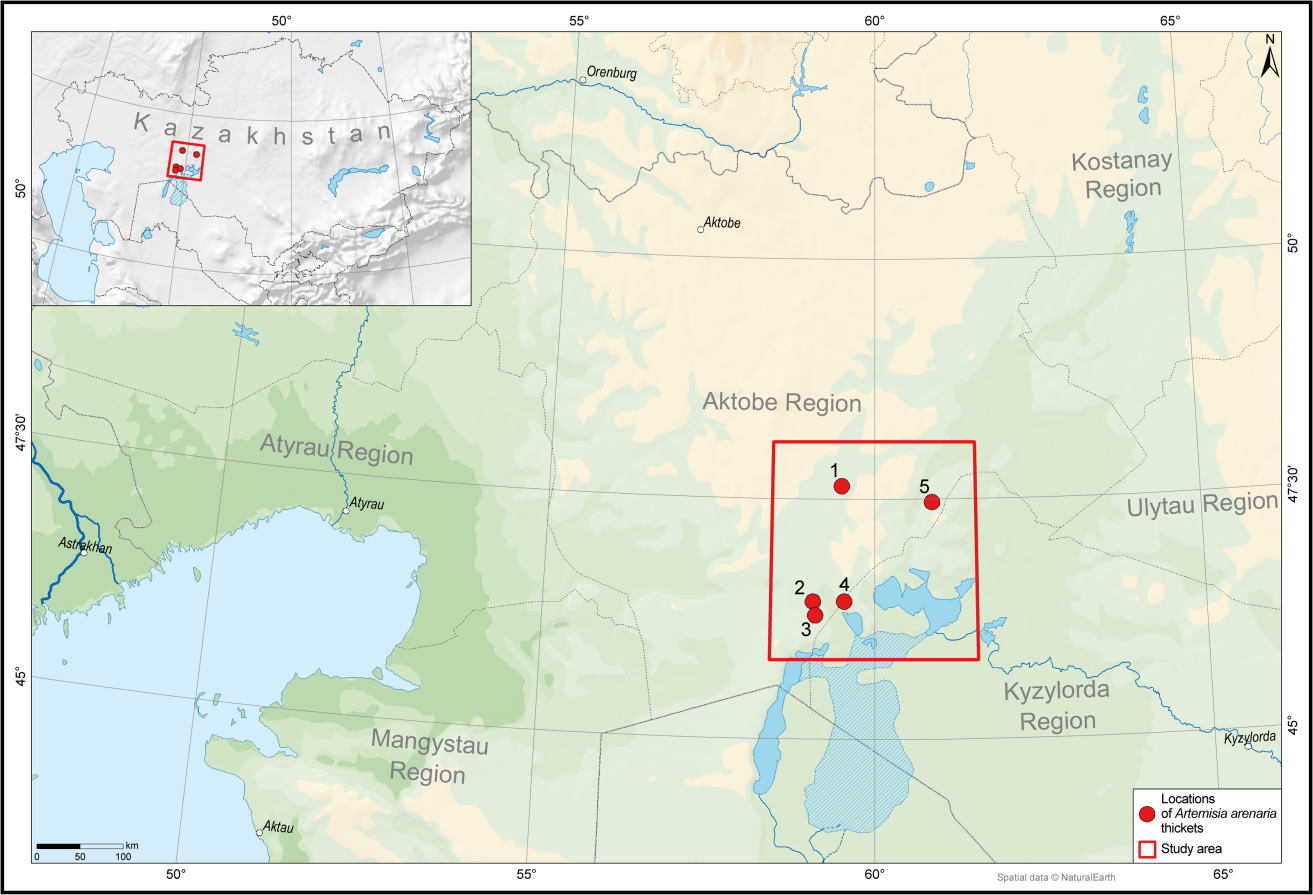
Locations of *Artemisia arenaria* thickets in the Northern Aral Sea region. The map was compiled with Natural Earth spatial data using QGIS software.

Field studies evaluating the raw material reserves of *A. arenaria* were carried out in the Shalkar district of the Aktobe region and the Aral district of the Kyzylorda region. These studies were conducted in the second part of August 2024, during the budding stage of species (Fig. 2). The budding stage was chosen because many *Artemisia* species accumulate the highest levels of essential oils and bioactive compounds at this phenological stage (Egeubaeva, 2002).

The regional resource survey was conducted in accordance with the standard (Methodology for determining reserves of medicinal plants, 1986). Geobotanical descriptions were undertaken at each site containing resource species (Bykov, 1978), and community descriptions were carried out using traditional geobotanical methods (Korchagin, 1964; Poniatovskaya, 1964). The area of small thickets (up to 50 ha) was determined in the field using a GPS device. For thickets larger than 50 ha, areas were determined using Sentinel-2 satellite imagery with a spatial resolution of 10 m and calculated in ArcGIS using the polygon area calculation tool.

### Geobotanical description

Vegetation surveys were conducted for each plant community by recording geographical position and environmental settings, including landscape features, soil characteristics, hydrological conditions and the degree of human impact. The vertical and horizontal structure of the community and its total projective coverage were determined. Floristic composition was recorded in detail with phenological stages, vigour (using a five-point scale), abundance (following the Drude scale), spatial distribution (according to Bykov’s scale), and key morphometric traits such as plant height and growth form. Total projective coverage was defined as the projection of vegetation onto the ground surface, expressed as the proportion of the plot area visually covered by aboveground phytomass. The projective coverage of *A. arenaria* was estimated as its percentage contribution to the total projective coverage of the community. Both measurements were made visually at the site level. The herbarium was collected. The taxonomic identification of vascular plants was performed using two volumes of the Goloskokov (1969) and Goloskokov (1972). The scientific names of plants are given in accordance with POWO (Royal Botanic Gardens Kew, 2024).

### Estimating the aboveground biomass (productivity)

The yield was determined using two basic methods: clipping (usually on square plots) (thicket 1) and model bushes (on transects) (thickets 2-5) (Brown, 1954; Ramensky et al., 1956; Bykov, 1978).

### Clipping method

The sampling design was based on classical geobotanical methodology and adapted to the structural characteristics of *A. arenaria* thickets. In thicket 1, the population was relatively homogeneous, with specimens of similar parameters of medium size. Therefore, biomass was estimated using the clipping method. The clipping method involves clipping aboveground plant material (standing crop—either the current year’s growth or the total) within a quadrat and weighing it. In this study, aboveground biomass included stems and leaves, excluding lignified parts and roots. Metal frames (quadrats) of 0.25 sq m were used to determine the standing crop (Bykov, 1957). The frames were placed objectively, preferably with randomisation, in a 12-fold repetition (total area of 3 sq m), a design shown to increase sampling precision by reducing quadrat size while increasing replication (Kondratyeva, 1935; Brown, 1957; Bykov & Golovina, 1965). On desert rangelands, phytomass is clipped at 1–5 cm above the soil surface. The phytomass samples were weighed in the field immediately after clipping, then were cut into small fragments and spread in a thin layer on kraft paper. The material was air-dried in a well-ventilated, shaded area at ambient temperature until a constant weight was reached (usually 7–10 days). This drying method is appropriate for preserving volatile compounds, as it minimizes thermal degradation and evaporation losses prior to GC-MS analysis.

### Model bushes method

In contrast, in thickets 2–5, *A. arenaria* specimens differed markedly in plant volume, representing large, medium, and small size groups. In these structurally heterogeneous thickets, we applied the model bushes method. This method is used to determine the productivity of shrub and semi-shrub vegetation. The method involves laying transect (100 sq m) on which all species are calculated, except for seedlings. The calculation was carried out by grouping plants into 2–3 groups with similar heights and diameters. On the transect, all specimens were allocated to one of three categories: large, medium, and small. Model plants of each size were selected, and the annual growth was cut off. As in the clipping method, the raw phytomass samples were weighed in the field, then dried to an air-dried state and weighed again.

All obtained phytomass data were entered into tables, and the yields were converted to centners or kilograms per hectare. Next, the operational stock (OS) was determined by multiplying the area of the thicket by the lower bound of yield according to the following formula:

(1) \begin{eqnarray*}OS=S\times (M-2m).\end{eqnarray*}

OS—operational stock of air-dried raw materials, t/ha;

S—the area occupied by the species, ha;

M—the yield, kg/ha;

m—standard error of the mean (SEM) calculated for the yield.

Then, taking into account the 4-year recovery period of the aboveground part of the perennial wormwood species, the volume of possible annual processed air-dried raw material (VPAP) was determined according to the following formula:

(2) \begin{eqnarray*}VPAP=OS/(4+1).\end{eqnarray*}

VPAP—the volume of possible annual processed air-dried raw material, t;

OS—operational stock of air-dried raw materials, t/ha.

### Natural essential oil extraction

Essential oil was isolated from the air-dried whole aerial parts of *A. arenaria* from a single population (thicket 2) by hydrodistillation using a Clevenger apparatus. The plant-to-water ratio was approximately 1:5–1:8 (*w/v*), and distillation was carried out for 1.5–3 h. From 170 g of air-dried aerial parts, 0.6 ml of essential oil was obtained, corresponding to a yield of 0.39%–0.63% (a.d.w.). The oil was dried over anhydrous sodium sulphate, and the yield was calculated as a percentage of the dry weight. GC-MS analysis of the essential oil was performed on an Agilent 7890-5975C system. Since the plant material was analysed from only one natural population, the resulting chemical profile reflects site-specific characteristics and does not permit assessment of intraspecific chemotypic variability.

### Gas chromatography—mass spectrometry analysis

The chemical composition of the essential oils was analysed using gas chromatography coupled with mass spectrometry (GC-MS). The analyses were carried out on an Agilent 7890A gas chromatograph equipped with a 5975C mass-selective detector and a DB-17ms capillary column (30 m × 0.25 mm, film thickness 0.25 µm). Helium (99.995% purity) was used as the carrier gas at a constant flow rate of 1.0 mL/min. Samples were injected in split mode (25:1) at a 1.0 µL injection volume. The injector temperature was maintained at 250 °C. The oven temperature programme was set as follows: initial temperature 40 °C (held for 3 min), increased at 5 °C/min to 250 °C, and held at 250 °C for 3 min. The transfer line, ion source, and quadrupole temperatures were 260 °C, 230 °C, and 150 °C, respectively. Electron impact ionisation (EI) at 70 eV was applied, and mass spectra were recorded in the m/z range 40–550.

The identification of compounds was tentative, based on comparison of the obtained mass spectra with the NIST17 library and calculated retention indices (RIs). Compound abundances were semi-quantitatively assessed based on relative peak areas. Chromatographic data were processed using Agilent MassHunter Unknowns Analysis (version 10.1) and manually verified peak assignments (Quimby, 2024).

## Results

### Phytocenotic and resource characteristics

**Figure 3:**
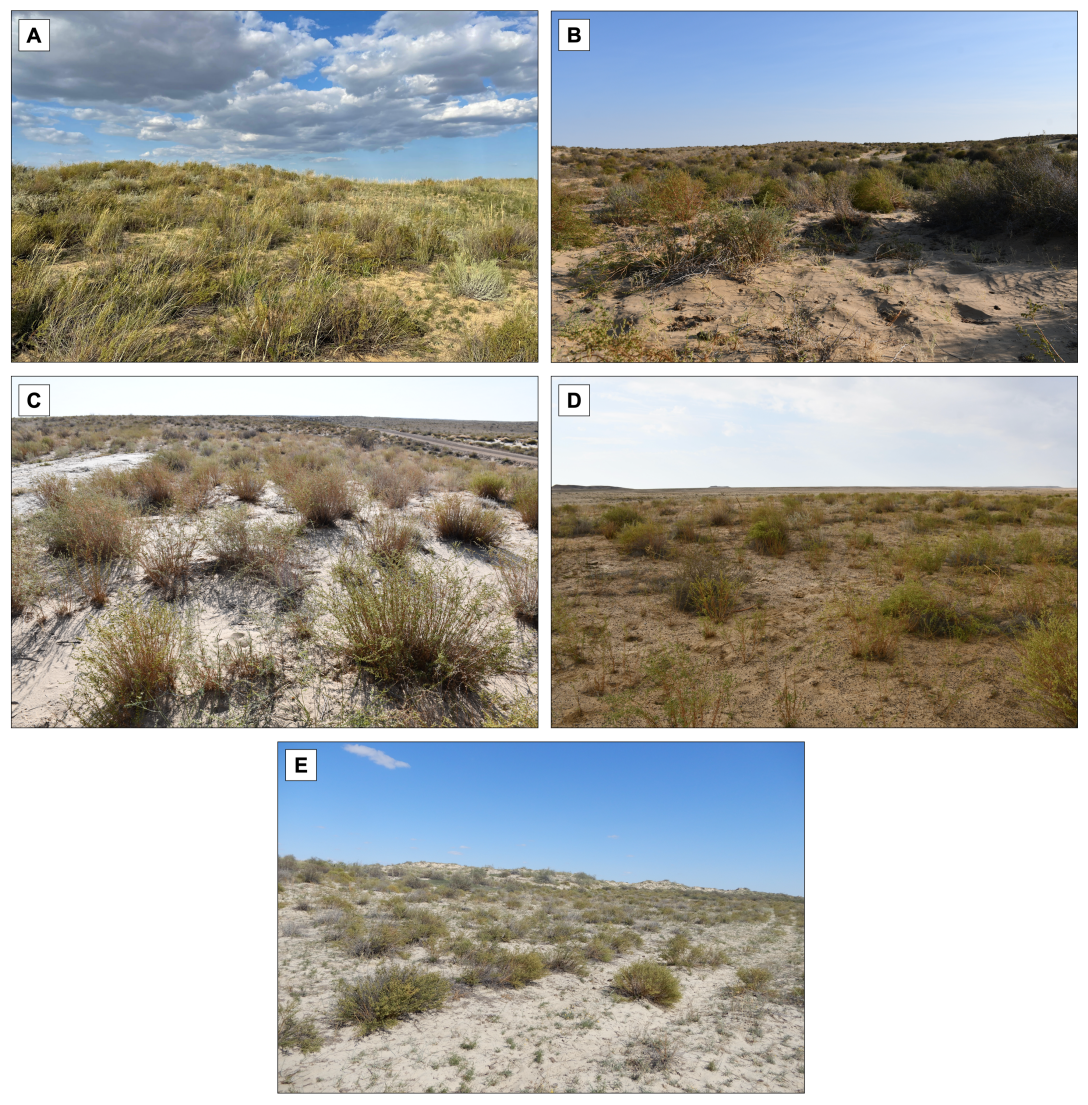
Identified thickets (1–5) of *Artemisia arenaria* in the Northern Aral Sea region. (A) population 1, (B) population 2, (C) population 3, (D) population 4, (E) population 5.

As a result of the field survey, five *A. arenaria* populations were identified, forming thickets ranging in area from 25.32 to 197.43 ha (Fig. 3).

It was established that, within the surveyed area, *A. arenaria* occurs in forb-wheat grass-wormwood, wormwood-shrub, forb-wormwood, ephemeral-wormwood, and grass-wormwood communities at altitudes of 201–256 m above sea level. The total projective coverage of communities ranged from 40 to 50%, while the projective coverage of *A. arenaria* in communities varied from 10 to 25%. *A. arenaria* was recorded over a total area of 464.73 ha, of which the species-occupied area did not exceed 51.8 ha.

The floristic composition of plant communities was determined for each site (Table 1). The species composition of the *A. arenaria* communities includes 29 species of vascular plants, among which are *Artemisia lercheana* Weber ex Stechm., *Alyssum desertorum* Stapf, *Calligonum aphyllum* (Pall.) Gürke, *Bassia prostrata* (L.) Beck, *Atraphaxis spinosa* (L.), *Poa bulbosa* L., *etc*.

**Table 1:** Characteristics of *Artemisia arenaria* thickets in the Northern Aral Sea region (August 2024).

Number of thickets	Coordinates	Location of the thickets	Plant community	Floristic composition of plant communities
1	47.639118° 59.491155°, *h* = 256 m	Aktobe Region, Shalkar District, near the village of Kyzylzhuldyz	Forb - wheat grass - wormwood	*Artemisia arenaria* DC.*, A. lercheana* Weber ex Stechm*., Agropyron fragile* (Roth) P.Candargy*, Helichrysum arenarium* (L.) Moench*, Erysimum siliculosum* (M.Bieb.) DC.*, Euphorbia seguieriana* Neck*., Leymus racemosus* (Lam.) Tzvelev*, Bassia prostrata* (L.) Beck
2	46.433501° 59.061836° *h* = 201 m	Aktobe Region, Shalkar District, Bolshye Barsuki Sands	Wormwood - shrub	*Artemisia arenaria, Atraphaxis spinosa* (L.)*, Krascheninnikovia ceratoides* (L.) Gueldenst.*, Artemisia lercheana, Eremurus inderiensis* (M.Bieb.) Regel*, Alhagi pseudalhagi* (M.Bieb.) Desv. ex Wangerin*, Centaurea pulchella* Ledeb*., Calligonum aphyllum* (Pall.) Gürke
3	46.290345° 59.104410° *h* = 210 m	Aktobe Region, Shalkar District, Bolshye Barsuki Sands	Forb - wormwood	*Artemisia arenaria, Bassia prostrata, Salsola paulsenii* Litv*., Artemisia lercheana, Carex pachystylis* J.Gay*, Poa bulbosa* L.*, Ceratocarpus arenarius* L.*, Atraphaxis spinosa, Alhagi pseudalhagi, Euphorbia seguieriana, Chondrilla ambigua* Fisch. ex Kar. & Kir.*, Allium caspium* (Pall.) M.Bieb*., Calligonum aphyllum*
4	46.436652° 59.540265° *h* = 237 m	Kyzylorda Region, Aral District, near the village of Kulandy	Ephemeral - wormwood	*Artemisia arenaria, A. lercheana, Atraphaxis spinosa, Krascheninnikovia ceratoides, Poa bulbosa, Eremurus inderiensis, Alyssum desertorum* Stapf*, Ceratocarpus arenarius, Centaurea pulchella, Ephedra intermedia* Schrenk & C.A.Mey*., Allium caspium*
5	47.469952° 60.878347° *h* = 245 m	Aktobe Region, Shalkar District, Malye Barsuki Sands	Grass - wormwood with *Calligonum aphyllum*	*Artemisia arenaria, A. lercheana, Agropyron fragile, Koeleria glauca* (Spreng.) DC.*, Bassia prostrata, Erysimum siliculosum, Alyssum desertorum, Tanacetum achilleifolium* (M.Bieb.) Sch.Bip.*, Poa bulbosa, Ephedra distachya* L*., Phragmites australis* (Cav.) Trin. ex Steud.*, Calligonum aphyllum, Cynanchica graveolens* subsp*. danilewskiana* (Basiner) P.Caputo & Del Guacchio*, Bromus tectorum* L*., Jurinea cyanoides* (L.) Rchb.

The yields of aboveground phytomass and operational stock, and the volume of possible annual air-dried raw materials, were also determined (Table 2). The yield of aboveground phytomass of *A. arenaria* varied from 164.0 ± 19.7 kg/ha (Aral district of the Kyzylorda region) to 585.0 ± 70.2 kg/ha (Shalkar district, Bolshye Barsuki Sands) for air-dried raw materials.

**Table 2:** Stocks of aerial phytomass of *Artemisia arenaria* in the Northern Aral Sea region.

Number of thickets	The area, ha		Yield of air-dried aboveground phytomass, kg/ha (mean ± SEM)	Operational stock of air-dried raw materials, metric tons	Volume of possible annual processed air-dried raw material, metric tons
	Total	Occupied by *Artemisia arenaria*			
1	51.76	10.35	453.0 ± 54.4	3.56	0.71
2	197.43	13.54	205.0 ± 24.6	2.11	0.42
3	25.32	4.81	585.0 ± 70.2	2.14	0.43
4	72.67	10.17	164.0 ± 19.7	1.27	0.25
5	117.55	12.93	405.0 ± 48.6	3.98	0.80
Total	464.73	51.8	–	13.06	2.61

**Figure 4:**
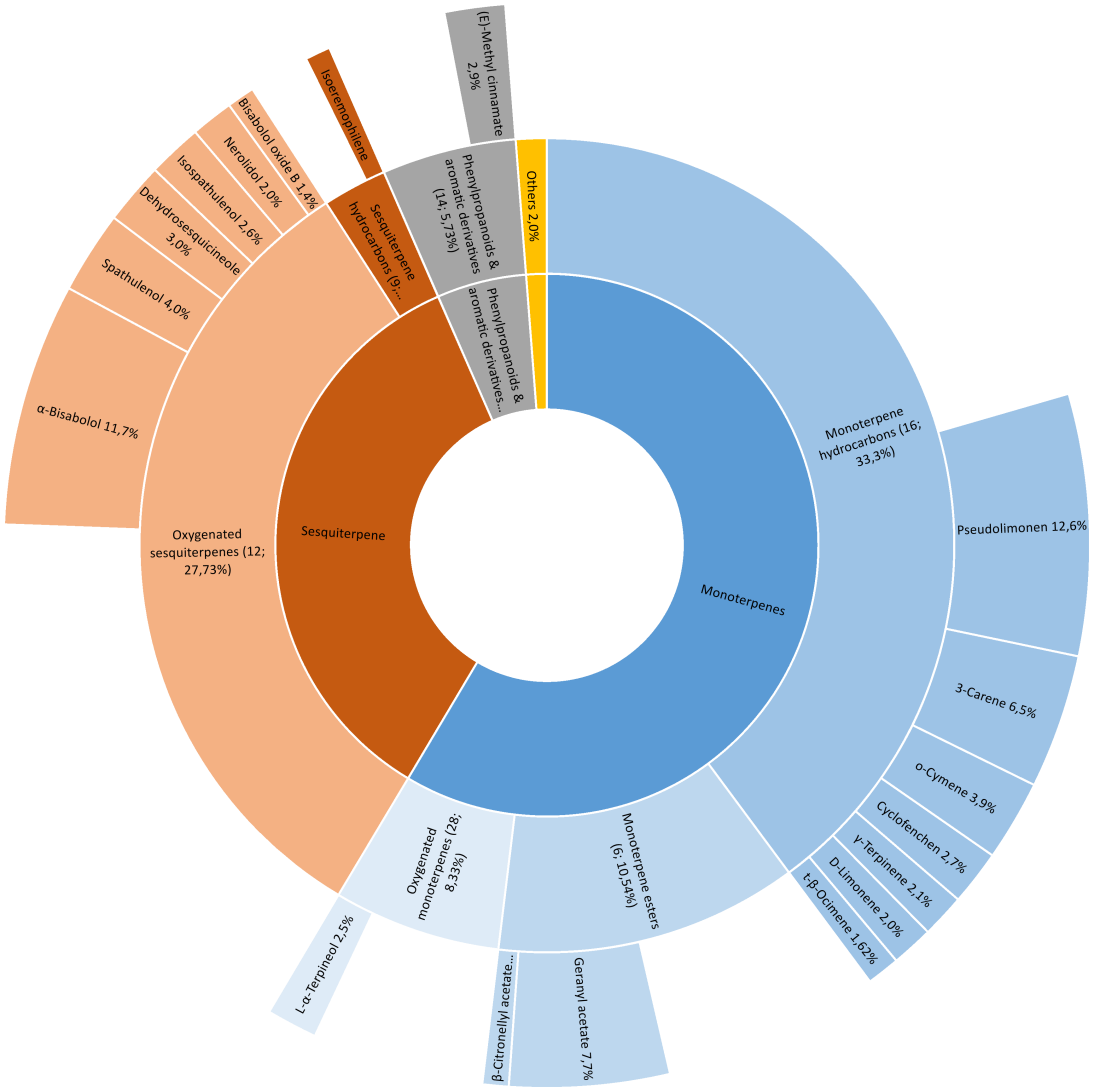
Dominant compounds of the essential oil of *Artemisia arenaria*.

Essential oil components of *A. arenaria* were identified by GC-MS using a match threshold of 85% or higher against the NIST 17 mass spectral library. Of the 149 detected peaks (95.5%), 105 met this criterion and were included in the analysis, representing most of the oil composition (90.5%). Eighteen compounds (approximately 70% of the total) were considered dominant (each with a relative abundance exceeding 1.0%) and are presented in Fig. 4. Peaks below the threshold (*n* = 42) were excluded and are expected to have minimal impact on the overall profile.

**Figure 5:**
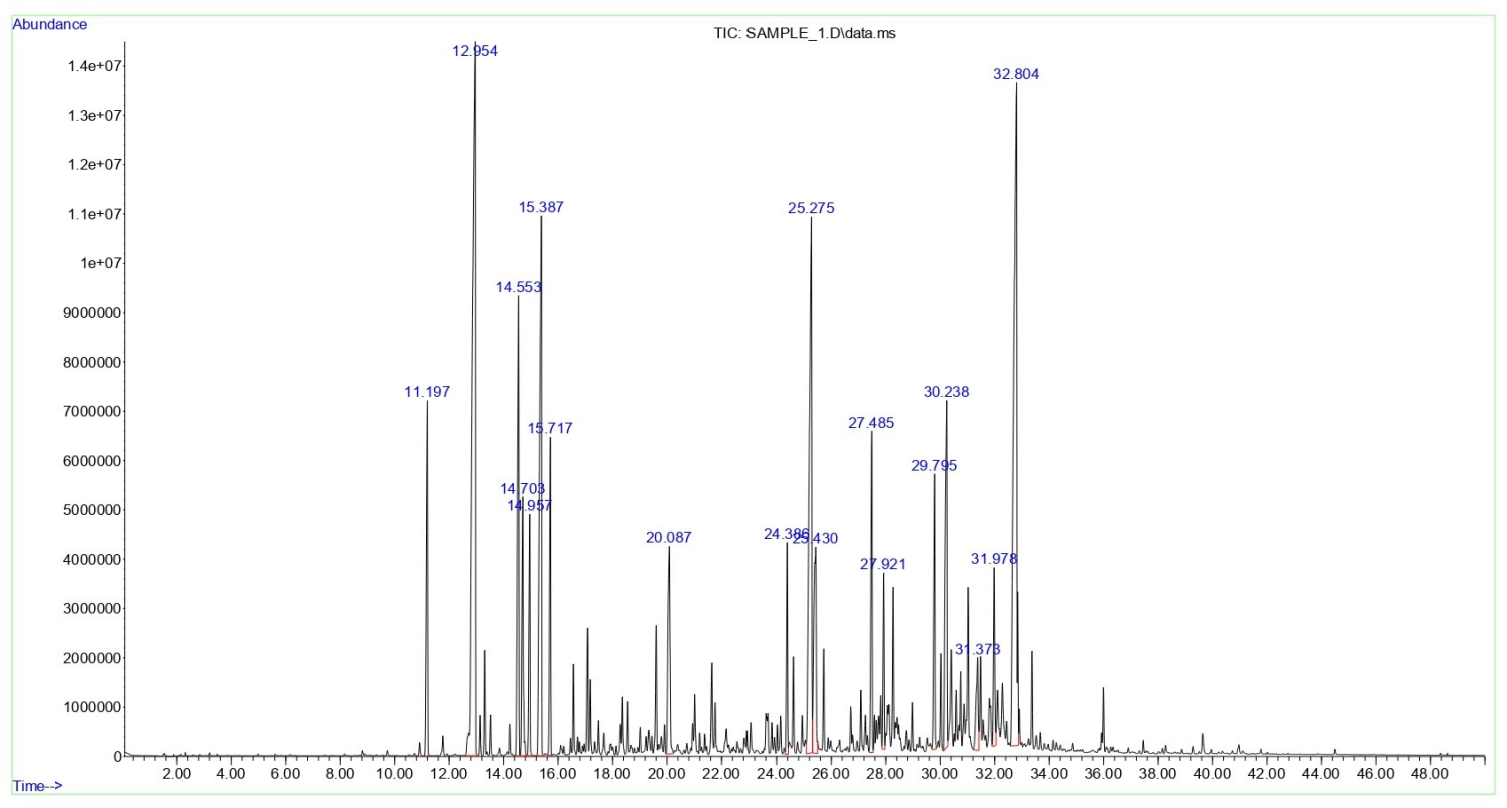
GC-MS chromatogra m of the essential oil of *Artemisia arenaria*.

The GC-MS chromatogram of *A. arenaria* essential oil is shown in Fig. 5, with numbered peaks corresponding to the compounds listed in Supplemental File 1. Percentage values were calculated based on relative peak areas.

The obtained GC-MS results are descriptive and provide an initial chemical profile of the analysed population of *A. arenaria*.

## Discussion

### Phytocenotic and resource characteristics

The assessment of the resource potential showed that, in five thickets dominated by *A. arenaria*, covering a total area of 51.8 ha, the total operational stock consisted of 13.06 tons of air-dried raw materials with a possible annual processed volume of 2.61 tons. Among these five thickets, two (1, 5), located in the vicinity of the village of Kyzylzhuldyz and in the Malye Barsuki Sands, are more productive and occupy larger areas. The possible annual processed volume from these thickets (0.71−0.8 tons) would not exceed 1.0 tons, taking into account the 4-year recovery period of the aboveground parts of wormwood.

The data were derived from yield assessments and plot area measurements, which ranged from 25.32 to 197.43 ha.

There is no available information on the reserves of raw materials in this region or in other territories where *A. arenaria* is present. Therefore, for comparison, we used rangeland yield data converted to kg/ha. For shrub-*Bassia*-psammophytic wormwood (*Artemisia arenaria, A. tomentella, A. santolina, Bassia prostrata, Calligonum aphyllum, Convolvulus fruticosus*) rangelands, the yield in summer was 250.5 kg/ha (Kurochkina & Osmanova, 1973), and the projective coverage of *A. arenaria* usually did not exceed 20%. The average yield of the five studied thickets was 362.4 kg/ha, excluding related species. This indicates that the yield of *A. arenaria* in 2024 exceeded the long-term average data, which may be due to several factors: a decrease in grazing intensity, climatic conditions in that year, an increase in the phytocenotic significance of the species, and the predominance of sand wormwood compared to other psammophylic species.

The floristic composition of the studied communities showed a consistent presence of sagebrush *Artemisia lercheana* from the section *Seriphidium* Besser ex W.Hook., as well as frequent occurrences of the shrubs *Calligonum aphyllum* and *Atraphaxis spinosa*. Indicator species of overgrazing were also recorded: *Euphorbia seguieriana, Ceratocarpus arenarius,* and *Eremurus inderiensis*. The presence of perennial grasses (*Agropyron fragile, Leymus racemosus, Koeleria glauca*) is typical for *A. arenaria* communities, while ephemers and ephemeroids such as *Alyssum desertorum, Poa bulbosa,* and *Bromus tectorum* occur only rarely (Makulbekova, 2003). Our research confirms these data, and we can further add *Erysimum siliculosum, Centaurea pulchella, Allium caspium*, and *Jurinea cyanoides* to the list of ephemeral species.

### GC-MS analysis

GC-MS analysis of the essential oil of *A. arenaria* revealed that the main classes of compounds were monoterpenes and sesquiterpenes. Among the abundant components were pseudolimonene (12.6%) and sesquiterpene alcohol α-bisabolol (11.77%). According to previous studies, α-bisabolol has been reported to exhibit anticancer, antinociceptive, neuroprotective, cardioprotective, antioxidant, and antimicrobial activities (Eddin et al., 2022; Ramazani et al., 2022). Ointments containing bisabolol have also been described in the literature as components of combination therapy for the local management of erosive and ulcerative oral mucosal lesions and for the prevention of cheilitis during inter-relapse periods (Allik et al., 2021; Varothai et al., 2024).

Significant amounts of geranyl acetate (7.74%), an ether characterised by sweet floral aroma, and the bicyclic monoterpene 3-carene (6.5%) were also detected in the present study. Geranyl acetate is widely used in cosmetics and perfumery and has been reported to possess antimicrobial and antifungal properties (Manjunath et al., 2023; Mohr Celuppi et al., 2023).

Other notable components included farnesol (2.06%) and pinene-type monoterpenes (2.67%). In total, 18 compounds were identified as dominant constituents forming the main profile of the essential oil (approximately 70% of the total composition). Overall, the composition of *A. arenaria* essential oil differed from previously published data, showing lower α-bisabolol and D-limonene levels and relatively higher proportions of oxygenated sesquiterpenes (Velikorodov et al., 2011). These variations may be associated with ecological factors, habitat conditions, and the plant developmental stage (Egeubaeva, 2002; Issayenko et al., 2022; Goel et al., 2019; Malaspina et al., 2025). Beyond environmental influences, differences in essential oil composition among *Artemisia* species also reflect evolutionary and genetic factors (Maffei, 2010; Brown, 2010). Volatile compounds in the plants are produced through secondary-metabolite pathways coded by nuclear, plastid, and mitochondrial genomes. Additionally, interspecific variation often results from allelic polymorphisms in terpene-biosynthetic and regulatory genes. Comparisons with other wormwood species native to Kazakhstan, which grow in steppe and desert areas, further demonstrate the chemical diversity within the genus. For instance, *A. scoparia* is dominated by α-/β-pinene (4.17/13.6%), β-ocimene (10.3%), myrtenol (3.5%), and methyl eugenol (27.5%), whereas *A. leucoides* shows a camphor-rich (58%) profile with notable amounts of 1,8-cineole, camphene, borneol, and α-bisabolol (1.05%) (Egeubaeva, 2002; Basher et al., 1997). Similar patterns were reported for *A. austriaca*, where hierarchical cluster analysis revealed two distinct chemotypes associated with geographic origin: a thujone-rich (32.5–21.6%) type and a camphor/1,8-cineole (13.9–7.8%) type. Cluster II was distinguished by the presence of camphor (40.5–17.4%) and 1,8-cineole (19.4–9.5%) (Issayenko et al., 2022).

The second group comprised 48 compounds with medium content (0.16–0.9%), including relatively high levels of oxygenated derivatives such as linalool (0.89%), terpinene-4-ol (0.85%), and caryophyllene oxide (0.29%). Methylsalicylate (0.14%) and thymol (0.22%) were also detected. In particular, the essential oil of the studied specimen contained comparatively higher amounts of oxygenated sesquiterpenes such as spathulenol (4.0%) and nerolidol (2.06%). Oxygenated monoterpenes frequently predominate in *Artemisia* oils (Goel et al., 2019), and as the environment shifts from mesic (moderately moist) to xeric (dry) habitats, both the concentration and structural diversity of these compounds in essential oils increase. However, in arid steppe and desert regions, the highest accumulation of sesquiterpenoids occurs in plants growing in localised moist microhabitats, such as depressions or riparian areas. In contrast, during summer water deficits, plants tend to allocate more metabolic resources to the production of monoterpene hydrocarbons and their oxygenated derivatives (Zhihitjapova et al., 2014).

Twenty-two compounds were isolated into a trace group (0.01–0.09%), where tremetone α- and β-unsaturated arylketone (benzofuran type) were identified, which have toxic properties and are related to specific secondary metabolites found in *A. campestris* (up to 15%) (Sura et al., 2012).

The predominance of pseudolimonene and α-bisabolol defines the chemical profile of the analysed essential oil of *A. arenaria* and may indicate chemotypic specificity of the studied population. This highlights the need for further studies across additional populations to assess the stability and variability of this pattern.

## Conclusions

In this study, the resource potential and phytochemical composition of the essential oil of *A. arenaria* were evaluated for the first time in the Northern Aral Sea region. The assessment of its yield in this region indicates that the studied thickets may represent a potential phytomass resource, providing a preliminary basis for further investigations.

GC-MS analysis showed that the essential oil of *A. arenaria* is characterised by pseudolimonene, α-bisabolol, geranyl acetate, and 3-carene as the major constituents. Several components detected in this study, including geranyl acetate, have not previously been reported for this species in the available literature. These findings expand current knowledge of its chemical composition. Further studies involving multiple populations, seasonal sampling, and biological assays are required to better assess the variability and biological potential of *A. arenaria* essential oil.

Given the wide distribution of this species in Kazakhstan, future research across different regions will be important for identifying regional characteristics and supporting the sustainable use of plant resources.

##  Supplemental Information

10.7717/peerj.21295/supp-1 Supplemental Information 1 Compounds identified by GC-MS analysis with mass spectral matches ≥85%

10.7717/peerj.21295/supp-2 Supplemental Information 2 Quantitative indicators of model plants (*Artemisia arenaria*)

10.7717/peerj.21295/supp-3 Supplemental Information 3 Raw and air-dried aboveground biomass of *Artemisia arenaria*

## Additional Information and Declarations

### Data Availability

The following information was supplied regarding data availability: The raw data are available in the Supplemental Files.
