# Supplementary material for: Resource potential and essential oil composition of Artemisia arenaria DC. in the Northern Aral Sea Region
Source: PeerJ. 2026 May 13;14:e21295. doi: 10.7717/peerj.21295 (PMC13179741; doi:10.7717/peerj.21295)
Supplement: Supplemental Information 1 [file peerj-14-21295-s001.docx]

| **RT** | **Compounds** | **Area %** |
| --- | --- | --- |
| **Monoterpene hydrocarbons (15)** | | **33.3** |
| 10.7 | Tricyclo[2.2.1.0(2,6)]heptane, 1,7,7-trimethyl- | 0.01 |
| 10.9 | Bicyclo[3.1.0]hex-2-ene, 2-methyl-5-(1-methylethyl)- | 0.09 |
| 11.2 | Tricyclo[2.2.1.0(2,6)]heptane, 1,3,3-trimethyl- (Tricyclene) | 2.67 |
| 11.8 | Camphene | 0.14 |
| 11.9 | Bicyclo[3.1.0]hex-2-ene, 4-methylene-1-(1-methylethyl)- | 0.01 |
| 12.7 | Bicyclo[3.1.0]hexane, 4-methylene-1-(1-methylethyl)- (Sabinene) | 0.28 |
| 13.0 | Cyclohexane, 1-methylene-4-(1-methylethenyl)- | 12.66 |
| 13.3 | Bicyclo[3.1.1]heptane, 6,6-dimethyl-2-methylene- (β-Pinene) | 0.60 |
| 14.2 | (+)-4-Carene | 0.18 |
| 14.6 | o-Cymene | 3.91 |
| 14.7 | D-Limonene | 2.03 |
| 15.0 | t-β-Ocimene | 1.62 |
| 15.4 | 3-Carene | 6.49 |
| 15.7 | γ-Terpinene | 2.10 |
| 16.6 | Cyclohexene, 1-methyl-4-(1-methylethylidene)- (Terpinolene) | 0.51 |
| **Oxygenated monoterpenes (27)** | | 8.33 (8.35) |
| 16.2 | 3-Oxatricyclo[4.1.1.0(2,4)]octane, 2,7,7-trimethyl- | 0.10 |
| 16.8 | 3-Methyl-2-(2-methyl-2-butenyl)-furan | 0.08 |
| 17.1 | Linalool | 0.89 |
| 17.2 | 1,5,7-Octatrien-3-ol, 3,7-dimethyl- | 0.45 |
| 17.7 | Fenchol | 0.17 |
| 17.8 | 2-Cyclohexen-1-ol, 1-methyl-4-(1-methylethyl)-, cis- | 0.02 |
| 17.9 | 1,7,7-Trimethylbicyclo[2.2.1]hept-5-en-2-ol | 0.11 |
| 18.4 | Bicyclo[3.1.1]heptan-3-ol, 6,6-dimethyl-2-methylene- | 0.41 |
| 18.6 | (+)-2-Bornanone (Camphor) | 0.28 |
| 18.7 | 2H-Pyran, 3,6-dihydro-4-methyl-2-(2-methyl-1-propenyl)- | 0.06 |
| 18.8 | Bicyclo[2.2.1]heptan-2-ol, 2,3,3-trimethyl- | 0.02 |
| 19.0 | Pinocarvone | 0.13 |
| 19.2 | Bicyclo[3.1.0]hex-3-en-2-one, 4-methyl-1-(1-methylethyl)- | 0.10 |
| 19.3 | endo-Borneol | 0.20 |
| 19.4 | Bicyclo[3.1.1]heptan-3-one, 2,6,6-trimethyl- | 0.08 |
| 19.6 | Terpinen-4-ol | 0.85 |
| 20.1 | L-α-Terpineol | 2.47 |
| 20.7 | 2-Cyclohexen-1-ol, 2-methyl-5-(1-methylethenyl)-, cis- | 0.06 |
| 20.9 | 2,6-Octadien-1-ol, 3,7-dimethyl-, (Z)- (Nerol) | 0.03 |
| 20.9 | Citronellol | 0.17 |
| 21.6 | Geraniol | 0.64 |
| 21.8 | 5-Cyclodecene, 1,2-epoxy- | 0.32 |
| 22.4 | 1-Cyclohexene-1-carboxaldehyde, 4-(1-methylethenyl)- | 0.06 |
| 22.6 | Thymol | 0.18 |
| 23.1 | Phenol, 2-methyl-5-(1-methylethyl)- (Carvacrol) | 0.24 |
| 23.5 | 2,4-Decadienal, (E,E)- | 0.02 |
| 24.1 | Bicyclo[3.2.1]octan-3-one, 6-(2-hydroxyethyl)-, endo- | 0.21 |
| **Monoterpene esters (6)** | | 10.54 |
| 23.6 | 2,6-Octadienoic acid, 3,7-dimethyl-, methyl ester | 0.34 |
| 23.7 | Myrtenyl acetate | 0.39 |
| 24.4 | 6-Octen-1-ol, 3,7-dimethyl-, acetate (Citronellyl acetate) | 1.27 |
| 24.6 | 2,6-Octadien-1-ol, 3,7-dimethyl-, acetate, (Z)- (Neryl acetate) | 0.53 |
| 24.9 | Cyclopentane, 1-acetoxymethyl-3-isopropenyl-2-methyl- | 0.27 |
| 25.3 | Geranyl acetate | 7.74 |
| **Sesquiterpene hydrocarbons (9)** | | 2.93 |
| 24.0 | Cyclohexene, 4-ethenyl-4-methyl-3-(1-methylethenyl)-1-(1-methylethyl)- | 0.14 |
| 25.9 | Naphthalene, 1,2,4a,5,8,8a-hexahydro-4,7-dimethyl-1-(1-methylethyl)- | 0.16 |
| 26.8 | Aromandendrene | 0.10 |
| 27.1 | Caryophyllene | 0.48 |
| 27.6 | Naphthalene, decahydro-4a-methyl-1-methylene-7-(1-methylethylidene)- | 0.19 |
| 27.8 | Benzene, 1-(1,5-dimethyl-4-hexenyl)-4-methyl- (ar-Curcumene) | 0.36 |
| 27.9 | Naphthalene, 1,2,3,5,6,7,8,8a-octahydro-1,8a-dimethyl-7-(1-methylethenyl)- | 1.23 |
| 28.5 | β-Bisabolene | 0.15 |
| 29.2 | Cyclohexene, 4-[(1E)-1,5-dimethyl-1,4-hexadien-1-yl]-1-methyl- | 0.12 |
| **Oxygenated sesquiterpenes (12)** | | 27.73 |
| 27.5 | (1R,3S,4S)-1,3-Dimethyl-3-(4-methylpent-3-en-1-yl)-2-oxabicyclo[2.2.2]oct-5-ene | 3.00 |
| 28.1 | 2-((2R,4aR,8aS)-4a-Methyl-8-methylenedecahydronaphthalen-2-yl)acrylaldehyde | 0.34 |
| 29.8 | 1,6,10-Dodecatrien-3-ol, 3,7,11-trimethyl- (Nerolidol) | 2.06 |
| 30.2 | 1H-Cycloprop[e]azulen-7-ol, decahydro-1,1,7-trimethyl-4-methylene- (Spathulenol) | 3.99 |
| 30.4 | Isospathulenol | 2.64 |
| 30.6 | Bergamotol, Z-.alpha.-trans- | 0.38 |
| 30.8 | Butanoic acid, 2-methyl-, 3,7-dimethyl-2,6-octadienyl ester | 0.45 |
| 30.9 | 5-Azulenemethanol, 1,2,3,4,5,6,7,8-octahydro-α,α,3,8-tetramethyl-, acetate | 0.35 |
| 31.5 | (-)-Spathulenol | 0.63 |
| 32.0 | Bisabolol oxide B | 1.36 |
| 32.3 | (E)-Valerenyl isovalerate | 0.76 |
| 32.8 | α-Bisabolol | 11.77 |
| **Phenylpropanoids and aromatic derivatives (14)** | | 5.73 |
| 12.2 | Benzaldehyde | 0.02 |
| 13.4 | Mesitylene | 0.02 |
| 16.7 | Benzene, 1-ethenyl-3,5-dimethyl- | 0.10 |
| 19.8 | Benzenemethanol, .alpha.,.alpha.,4-trimethyl- | 0.12 |
| 19.9 | Methyl salicylate | 0.14 |
| 21.0 | Benzene, 2-methoxy-4-methyl-1-(1-methylethyl)- | 0.40 |
| 24.5 | 3-Allyl-6-methoxyphenol | 0.09 |
| 25.4 | 2-Propenoic acid, 3-phenyl-, methyl ester, (E)- (Methyl cinnamate) | 2.93 |
| 25.7 | Methyleugenol | 0.58 |
| 26.7 | Benzoic acid, 2-methylbutyl ester | 0.26 |
| 28.1 | Benzene, 1,2-dimethoxy-4-(1-propenyl)- (Methyl isoeugenol) | 0.34 |
| 30.0 | 6-Octen-1-ol, 3,7-dimethyl-, benzoate | 0.59 |
| 33.7 | Tremetone | 0.09 |
| 34.4 | Benzyl Benzoate | 0.05 |
| **Fatty acids** | |  |
| 38.3 | n-Hexadecanoic acid | 0.06 |
| **Long-chain hydrocarbons** | |  |
| 41.0 | Heneicosane | 0.08 |
| 44.5 | Tricosane | 0.03 |
| **Aliphatic compounds (19)** | | 1.82 |
| 2.3 | 3-Buten-2-ol, 2-methyl- | 0.01 |
| 3.2 | 1-Heptene | 0.01 |
| 8.2 | 2-Hexenal, (E)- | 0.01 |
| 8.8 | 1-Hexanol | 0.03 |
| 8.9 | 3-Methoxy-3-methylbutanol | 0.01 |
| 9.7 | 5,10-Dioxatricyclo[7.1.0.0(4,6)]decane | 0.03 |
| 13.1 | 5-Hepten-2-one, 6-methyl- | 0.22 |
| 13.5 | 9-Oxabicyclo[6.1.0]nonane | 0.23 |
| 14.1 | Acetic acid, hexyl ester | 0.02 |
| 16.1 | Ethyl 2-(5-methyl-5-vinyltetrahydrofuran-2-yl)propan-2-yl carbonate | 0.08 |
| 17.0 | 6-Nonenal, (Z)- | 0.07 |
| 17.3 | Butanoic acid, 3-methyl-, 2-methylbutyl ester | 0.11 |
| 17.5 | (E)-4,8-Dimethylnona-1,3,7-triene | 0.20 |
| 18.3 | Bicyclo[3.1.1]heptan-2-one, 6,6-dimethyl- | 0.22 |
| 18.9 | 2-Nonenal, (E)- | 0.02 |
| 21.2 | cis-3-Hexenyl isovalerate | 0.10 |
| 21.4 | Butanoic acid, 3-methyl-, hexyl ester | 0.10 |
| 23.8 | Hexyl (E)-2-methylbut-2-enoate | 0.16 |
| 27.6 | Bicyclo[4.3.0]nonan-2-one, 8-isopropylidene- | 0.19 |
