# Supplementary material for: Resource potential and essential oil composition of Artemisia arenaria DC. in the Northern Aral Sea Region
Source: PeerJ. 2026 May 13;14:e21295. doi: 10.7717/peerj.21295 (PMC13179741; doi:10.7717/peerj.21295)
Supplement: Supplemental Information 2 [file peerj-14-21295-s002.docx]

Quantitative indicators of model plants (*Artemisia arenaria*)

| Model plants | Parameters of model plants (sq. m) | The density on the site 10x10 sq. m | The amount of plants per ha |
| --- | --- | --- | --- |
| 1 | 2 | 3 | 4 |
| Thicket 2 | | | |
| Large | 0.72х1.41х1.38 | 2 | 200 |
| Medium | 0.72х0.54х0.43 | 7 | 700 |
| Small | 0.49х0.28х0.28 | 3 | 300 |
| Thicket 3 | | | |
| Large | 0.80х1.42х0.83 | 6 | 600 |
| Medium | 0.72х0.73х0.66 | 13 | 1300 |
| Small | 0.60х0.50х0.44 | 30 | 3000 |
| Thicket 4 | | | |
| Large | 0.80х1.00х0.8 | 8 | 800 |
| Medium | 0.54х0.54х0.52 | 9 | 900 |
| Small | 0.54х0.3х0.3 | 48 | 4800 |
| Thicket 5 | | | |
| Large | 0.52х1.0х0.8 | 8 | 800 |
| Medium | 0.46х0.65х0.55 | 18 | 1800 |
| Small | 0.4х0.3х0.3 | 65 | 6500 |

Note: In the first population (thicket 1) of *Artemisia arenaria,* the clipping method (without model plants) was used as all specimens were of similar parameters of medium size.
