# Supplementary material for: Resource potential and essential oil composition of Artemisia arenaria DC. in the Northern Aral Sea Region
Source: PeerJ. 2026 May 13;14:e21295. doi: 10.7717/peerj.21295 (PMC13179741; doi:10.7717/peerj.21295)
Supplement: Supplemental Information 3 [file peerj-14-21295-s003.docx]

Raw and air-dried aboveground biomass of *Artemisia arenaria*

| Number of thickets | The name of the community | General projective coverage (%) | Primary biomass | Raw weight of aboveground part (g) | Air-dry weight of aboveground part (g) | Shrinkage (%) |
| --- | --- | --- | --- | --- | --- | --- |
| 1 | Forb - wheat grass - wormwood | 40 | 3 sq. m | 325 | 136 | 41.8 |
| 2 | Wormwood - shrub | 30 | 3 model plants | 1225 | 552 | 45.1 |
| 3 | Forb - wormwood | 35 | 3 model plants | 1395 | 646 | 46.3 |
| 4 | Ephemeral - wormwood | 35 | 3 model plants | 305 | 122 | 40 |
| 5 | Grass - wormwood with *Calligonum aphyllum* | 50 | 3 model plants | 385 | 224 | 58.2 |
